# Study on Hesitant Fuzzy Information Measures and Their Clustering Application

**DOI:** 10.1155/2019/5370763

**Published:** 2019-03-03

**Authors:** Jin-hui Lv, Si-cong Guo, Fang-fang Guo

**Affiliations:** ^1^Institute of Intelligence Engineering and Mathematics, Liaoning Technical University, Fuxin 123000, China; ^2^School of Economics and Management, Huainan Normal University, Huainan 232038, China

## Abstract

At present, research on hesitant fuzzy operations and measures is based on equal length processing, and an equal length processing method will inevitably destroy the original data structure and change the data information. This is an urgent problem to be solved in the development of hesitant fuzzy sets. Aiming at solving this problem, this paper firstly defines a hesitant fuzzy entropy function as the measure of the degree of uncertainty of hesitant fuzzy information and then proposes the concept of hesitant fuzzy information feature vector. The hesitant fuzzy distance measure and similarity measure are studied based on the information feature vector. Finally, the hesitant fuzzy network clustering method based on similarity measure is given, and the effectiveness of our algorithm through a numerical example is illustrated.

## 1. Introduction

Torra and Narukawa [1, 2] extended fuzzy sets [3] to hesitant fuzzy sets (HFSs) because they found that, under a group setting, it is difficult to determine the membership of an element to a set due to doubts between a few different values. For example, two DMs discuss the membership degree of *x* into *A*. One wants to assign 0.4 and the other 0.6, and they cannot persuade with each other; thus the membership degrees of *x* into *A* can be represented by {0.4, 0.6}. This is obviously different from fuzzy number 0.4 (or 0.6) and the intuitionistic fuzzy number (0.4, 0.6). Therefore, hesitant fuzzy sets can better simulate the hesitant preferences of decision-makers. Since it was put forward, the hesitant fuzzy set has received extensive attention from scholars at home and abroad. The main research work is concentrated in the following aspects: (1) research on various measures in the hesitant fuzzy environment [4–10]; (2) research on the integration operator of hesitant fuzzy information [11–16]; and (3) the expansion of hesitant fuzzy set theory [17–22].

It should be pointed out that the present researches on the operation, sorting, and various measures of hesitant fuzzy sets require that the hesitant fuzzy elements have the same length. In practical application, the length of hesitant fuzzy element is different. The method proposed in [2] is adding some elements to a shorter hesitant fuzzy element, making it equal to another hesitant fuzzy element, or repeating their elements in order to obtain two series with the same length [23]. Obviously, these methods will destroy the original data structure and change the data information. How to overcome the shortcomings has become an urgent problem to be solved in the development process of hesitant fuzzy sets.

Clustering is a basic technique, which is often utilized in a primary step of analyzing unlabeled data with the goal of summarizing structural information [24]. In practical applications, the clustering data are mostly uncertain or fuzzy. To solve the problem of data clustering in different fuzzy environments, fuzzy clustering algorithms [25], intuitionistic fuzzy clustering algorithms [26], and 2-type fuzzy clustering algorithms [27] have been proposed. However, in the group of decision-making environment, the decision information is more suitable to express hesitant fuzzy sets, and the algorithm mentioned above is not suitable for handling the clustering problem of this type of information. If the fuzzy logic is used to handle it, generally take the average value of preference information that are provided by experts or can take the minimum range containing all of the preference information, that is, convert the hesitant fuzzy information into interval value information for processing. This method of data processing is bound to change the original preference information that provided by experts; as a result, the research of clustering problem under the hesitant fuzzy information has a certain scientific significance. One of the advantages of applying the hesitant fuzzy set is that clustering hesitant and vague information permits us to find patterns among hesitant fuzzy data. At present, the clustering researches under the hesitant fuzzy environment are still at the its initial stage, and Chen et al. [28] used the correlation coefficient of hesitant fuzzy set to construct hesitant fuzzy relationship matrix and then conducted hesitant fuzzy clustering analysis based on the relation of equivalence. In order to obtain an equivalence relation matrix, a fuzzy relation matrix needs to be iterated continuously, which not only loses information but also has a large amount of calculation [29]. Due to the existence of uncertainty for the similarity measure of samples, leading to the clustering, results were not precise enough and the divided categories were inconsistent with the fact. In [4], the hesitant fuzzy similarity measure formula based on distance was proposed. The measurement is inconsistent with the facts sometimes, and the resolution is not high enough; in the literature [29], a hesitant fuzzy clustering method based on agglomerative hierarchical clustering [30] was proposed. This method needs to use a hesitant fuzzy average operator to calculate the clustering center repeatedly, and the calculation amount is large; in the literature [31], a hesitant fuzzy clustering algorithm based on minimal spanning tree was proposed. The distance of hesitant fuzzy set used in this method is put forward based on the literature [4], which also has the shortcoming of low resolution and sometimes inconsistent with the fact; in the literature [32], from the point of view of information theory, hesitant fuzzy relative entropy and symmetric interactive entropy are proposed, a new kind of hesitant fuzzy similarity degree is proposed, which is combined with the idea of TOPSIS, and a hesitant fuzzy clustering method is proposed based on the traditional netting clustering method. The premise of all the above methods in the measurement and operation is that the data are equal in length, which is not satisfied by the hesitant fuzzy set. Therefore, it is necessary to add artificial elements for equal length processing, and the processed data will inevitably change the original data information and affect the clustering results.

Based on the above analysis, this paper firstly proposes the concept of hesitant fuzzy entropy function and hesitant fuzzy information feature vector, aiming at solving the problem of processing data of hesitant fuzzy set, sorting, and various measures in the study of different lengths. Furthermore, the hesitant fuzzy uncertainty measure, distance measure, and similarity measure are studied. Finally, based on the similarity measure and the traditional network clustering method, the network clustering method for hesitant fuzzy information is given. And then we illustrate its effectiveness via numerical examples.

## 2. Preliminary

**Definition 1 (1, 2).**

Let *X*={*x*_1,_*x*_2_,…, *x*_*m*_} be a fixed set; a hesitant fuzzy set (HFS) on *X* is represented by a function that when applied to *X*, it returns a subset of [0, 1], which can be expressed by a mathematical symbol:

(1) $$\begin{array}{c} H=\left\{<x,h_{H}\left(x\right)>\;|\;x\in X\right\}, \end{array}$$

where *h*_*H*_(*x*) is a set of some values in [0,1], denoting the possible membership degrees of the element *x* ∈ *X* to the set *H*. *h*_*H*_(*x*) is called the hesitant fuzzy number or hesitant fuzzy element. If it does not cause confusion, it can be abbreviated as *h*=*h*_*H*_(*x*). The hesitant fuzzy number can be expressed in more detail as *h*=*H*{*γ*^1^, *γ*^2^,…, *γ*^*l*^}. Among which, *l* denotes the number of elements in a hesitant fuzzy number *h*. Obviously, when *l*=1, the hesitant fuzzy set *H* degenerates into the traditional fuzzy set.

**Definition 2 (1, 2).**

Set *X*={*x*_1,_*x*_2_,…, *x*_*m*_} as a given nonempty set, then *H*^*c*^={<*x*, *h*_*H*_^*c*^(*x*) > ∣*x* ∈ *X*} is the complement of the hesitant fuzzy set *H*, among which

(2) $$\begin{array}{c} h_{H}^{c}\left(x\right)=\underset{\gamma \in h_{H}\left(x\right)}{⋃}\left\{1-\gamma \right\}. \end{array}$$

Distance measure and similarity measure are important research contents in fuzzy set theory and have a wide application background. In the literature [4], the axiomatic definitions of distance and similarity measure of hesitant fuzzy sets are given.

**Definition 3 (4).**

Sets *A*, *B* be the two hesitant fuzzy sets defined on *X*={*x*_1_, *x*_2_,…, *x*_*m*_}, and then the distance measure between *A* and *B* satisfies the following conditions:

1. 0 ≤ *d*(*A*, *B*) ≤ 1
2. *d*(*A*, *B*)=0, if and only if *h*_*A*_(*x*)=*h*_*B*_(*x*),  ∀ *x* ∈ *X*
3. *d*(*A*, *B*)=*d*(*B*, *A*)

**Definition 4 (4).**

Sets *A*, *B* be the two hesitant fuzzy sets defined on *X*={*x*_1_, *x*_2_,…, *x*_*m*_}, and then the similarity measure between *A* and *B* satisfies the following conditions:

1. 0 ≤ *S*(*A*, *B*) ≤ 1
2. *S*(*A*, *B*)=1, if and only if *h*_*A*_(*x*)=*h*_*B*_(*x*),  ∀ *x* ∈ *X*
3. *S*(*A*, *B*)=*S*(*B*, *A*)

Definition 2.3 is proposed to facilitate the use of distance measures to define similarity measures. In practice, the distance can only be satisfied with *d*(*A*, *B*) ≥ 0.

## 3. A New Kind of Hesitant Fuzzy Entropy

Entropy is the measurement of the degree of uncertainty of information, and it has always been an important research object in uncertainty decision analysis. A new hesitant fuzzy entropy measure function is proposed by analyzing the shortcomings in the current research results on hesitant fuzzy entropy.

**Definition 5 .**

Assign the hesitant fuzzy element *h*=∪_*γ*∈*h*_{*γ*}={*γ*_*j*_}_*j*=1_^*l*^, where *l* is the number of element in the hesitant fuzzy element, and record

(3) $$\begin{array}{c} x=\frac{1}{l}\overset{l}{\underset{j=1}{\sum }}\left(1-2\left|\gamma_{j}-0.5\right|\right),\\ y=\left\{\begin{array}{cc} \frac{2}{l\left(l-1\right)}\overset{l}{\underset{i,j=1,i<j}{\sum }}\left|\gamma_{i}-\gamma_{j}\right|, & l>1,\\ 0, & l=1, \end{array}\right. \end{array}$$

where *x* represents the fuzzy degree of the hesitant fuzzy element *h* and *y* represents the hesitant degree of the hesitant fuzzy element *h*. Then the real valued function *E* : *H*⟶[0,1] on the hesitant fuzzy element *h* can be expressed by a binary function *E*(*x*, *y*), if the *E*(*x*, *y*) meets the following conditions:

1. *E*(*x*, *y*)=0 if and only if *x*=0 and *y*=0
2. *E*(*x*, *y*)=1 if and only if (*x*, *y*)=(1,0) or (*x*, *y*)=(0,1)
3. ∂*E*/∂*x* > 0 and ∂*E*/∂*y* > 0, ∂^2^*E*/∂*x*^2^ > 0 and ∂^2^*E*/∂*y*^2^ > 0
4. *E*(*x*, *y*)=*E*(*y*, *x*)

Then, *E*(*x*, *y*) can be called as a hesitant fuzzy entropy function.

### 3.1. Interpretation and Analysis

1. *y*=0⟷*l*=1, *x*=0⟷*h*={0} ∨ *h*={1} ∨ *h*={0,1}. Then *x*=0 ∧ *y*=0⟷*h*={0} ∨ *h*={1} indicates that *h* is a clear set, then the entropy is 0.
2. When *l* > 1, since *x*=(1/*l*)∑_*j*=1_^*l*^(1 − 2|*γ*_*j*_ − 0.5|)=(1/*l*(*l* − 1))∑_*i*,*j*=1,*i*<*j*_^*l*^[(1 − 2|*γ*_*i*_ − 0.5|)+(1 − 2|*γ*_*j*_ − 0.5|)], *y*=(2/*l*(*l* − 1))∑_*i*,*j*=1,*i*<*j*_^*l*^|*γ*_*i*_ − *γ*_*j*_|=(2/*l*(*l* − 1))∑_*i*,*j*=1,*i*<*j*_^*l*^|(*γ*_*i*_ − 0.5)+(0.5 − *γ*_*j*_)| ≤ (2/*l*(*l* − 1))∑_*i*,*j*=1,*i*<*j*_^*l*^|*γ*_*i*_ − 0.5)|+|0.5 − *γ*_*j*_|, then it can get *x*+*y* ≤ 1, and it is concluded that the domain of the entropy function *E*(*x*, *y*) is {(*x*, *y*) | *x* ≥ 0, *y* ≥ 0, *x*+*y* ≤ 1} because the entropy function *E*(*x*, *y*) is concave increase with respect to *x* and *y*, and the maximum value of *E*(*x*, *y*) is 1 when (*x*, *y*)=(1,0) or (*x*, *y*)=(0,1) is obtained; that is, when *h*={0.5} or *h*={0,1}, the uncertainty reaches the maximum. *h*={0,1} is completely contradictory information, and *h*={0.5} is completely fuzzy information; in both cases, the uncertainty is maximized and in line with intuitive judgment.
3. It ensures that the entropy function is concavely increased with respect to fuzziness and hesitation degree, conforms to human cognitive characteristics, and improves discrimination.
4. Fuzziness and hesitancy have the same effect on entropy.

Based on the above analysis, function *E*(*x*, *y*)=*x*^2^+*y*^2^ obviously satisfies the above conditions in Definition 3.1, so it can be regarded as an entropy function. For example, if *h*={0.5}⟶*x*=1, *y*=0, then *E*(*h*)=*E*(*x*, *y*)=*E*(1,0)=1; if *h*={0,1}⟶*x*=0, *y*=1, then *E*(*h*)=*E*(*x*, *y*)=*E*(0,1)=1; and if *h*_1_={0.2}⟶*x*=0.4, *y*=0, *h*_1_={0.1, 0.2, 0.3}⟶*x*=0.4, *y*=0.13, then *E*(*h*_1_)=*E*(0.4, 0)=0.16, *E*(*h*_2_)=*E*(0.4, 0.13)=0.16+0.017=0.177, where *E*(*h*_1_) < *E*(*h*_2_). The above judgment results are consistent with the intuition.

**Property 1 .**

Set hesitant fuzzy element *h*=∪_*γ*∈*h*_{*γ*}={*γ*_*j*_}_*j*=1_^*l*^, when *l*=1; the hesitant fuzzy element *h* degenerates into a fuzzy number, and the entropy of fuzzy value *h* is *E*(*h*)=*E*(*x*, *y*).

**Proof**

1. *E*(*h*)=*E*(*x*, *y*)=0⟺*x*=0, *y*=0, that is, *h*={0} or *h*={1}, where *h* is a clear set.
2. According to condition (2) *E*(*h*)=*E*(*x*, *y*)=1⟺(*x*, *y*)=(0,1) or (*x*, *y*)=(1,0) because when *l*=1, *y*=0, so *E*(*h*)=*E*(*x*, *y*)=1⟺(*x*, *y*)=(1,0), that is, *h*={1/2}.
3. According to condition (3), it is known that *E*(*x*, *y*) increases monotonously with respect to *x*, so when *γ*_*j*_ is closer to 0.5, the larger the *x*=(1/*l*)∑_*j*=1_^*l*^(1 − 2|*γ*_*j*_ − 0.5|) is, the larger the entropy *E*(*h*)=*E*(*x*, *y*) of the fuzzy value *h* is.

The property 3.1 indicates that the fuzzy entropy is a special case of the hesitant fuzzy entropy function, and the hesitant fuzzy entropy function can also be applied to the fuzzy set.

In order to illustrate the advantage of the entropy function proposed in this paper in measuring uncertainty, the following is compared with the existing entropy formula: at present, the common formulas of hesitating fuzzy entropy include the entropy formula proposed by Xu and Xia and the entropy formula proposed by Farhadinia, in which

(4) $$\begin{array}{c} E_{\text{Xu}}\left(h\right)=-\frac{1}{l\;\ln\;2}\overset{l}{\underset{i=1}{\sum }}\left(\frac{\gamma^{i}+\gamma^{l-i+1}}{2}\ln\frac{\gamma^{i}+\gamma^{l-i+1}}{2}+\frac{2-\gamma^{i}-\gamma^{l-i+1}}{2}\ln\frac{2-\gamma^{i}-\lambda^{l-i+1}}{2}\right), \end{array}$$

where *l* indicates the number of elements in a hesitant fuzzy number *h* and *γ*^*i*^ indicates the element of the largest *i*th in the hesitant fuzzy number *h*.

(5) $$\begin{array}{c} E_{\text{Pa}}\left(h\right)=\frac{Z\left(2\;\;d\left(h,\left\{0.5\right\}\right)\right)-Z\left(1\right)}{Z\left(0\right)-Z\left(1\right)}, \end{array}$$

where *Z* : [0,1]⟶[0,1] is strictly monotonically decreasing function, which may get *Z*(*t*)=1 − *t*, *Z*(*t*)=(1 − *t*)/(1+*t*), *Z*(*t*)=1 − *t*^2^, *Z*(*t*)=1 − *te*^*t*−1^; *d*(*h*, {0.5})=(1/*l*)∑_*i*=1_^*l*^|*γ*^*i*^ − 0.5| (*γ*^*i*^ ∈ *h*, where *l* indicates the number of elements contained in the fuzzy number *h*).

Set hesitant fuzzy number *h*_1_=*H*{0.2, 0.4}, *h*_2_=*H*{0.3, 0.5}, *h*_3_=*H*{0.1, 0.2, 0.3}, *h*_4_=*H*{0.4, 0.5, 0.6}, *h*_5_=*H*{0.3, 0.6}, *h*_6_=*H*{0.4, 0.5}, *h*_7_=*H*{0.3, 0.5, 0.6}, and *h*_8_=*H*{0.2, 0.5, 0.7}. The entropy formula proposed by Xu and Xia and the entropy formula proposed by Farhadinia are compared with the entropy function proposed in this paper. The results are shown in Table 1.

Because the entropy formula proposed by Farhadinia only considers fuzziness and neglects the influence of hesitancy, the result is quite different from that of the method proposed in this paper and the method proposed by Xu. It is not difficult to find from the above table that the method proposed in this paper is obviously higher in the discrimination than that proposed by Xu, and the comparison result is close to it, and the individual results are inconsistent. For example, *E*_Xu_(*h*_4_) > *E*_Xu_(*h*_6_); however, according to the method presented in this paper, the result is *E*_Lv_(*h*_4_) < *E*_Lv_(*h*_6_); this is because the starting point is inconsistent and the hesitant fuzzy entropy proposed by Xu requires that the number of elements contained in the two pairs be equal and that the elements should be artificially added when the number of elements is different. Therefore, the proposed method is bound to deviate from intuitive judgment for the comparison of entropy of two hesitant fuzzy numbers with a different number of elements contained. The entropy measure function proposed in this paper not only considers the influence of fuzziness on the entropy value but also considers the effect of hesitation degree on the entropy value, which can more reasonably depict the uncertainty degree of the hesitation fuzzy number, so the result is more consistent with our intuition.

## 4. Hesitant Fuzzy Distance Measure and Similarity Measure

For a hesitant fuzzy element *h*=∪_*γ*∈*h*_{*γ*}={*γ*_*j*_}_*j*=1_^*l*^, the most important information it contains is the size of *γ* value and the degree of uncertainty, which is also a common concern in practical applications. Based on this, we introduce the definition of feature vector of hesitant fuzzy information.

**Definition 6 .**

Set hesitant fuzzy element *h*=∪_*γ*∈*h*_{*γ*}={*γ*_*j*_}_*j*=1_^*l*^; two-dimensional vector (*s*(*h*), *E*(*h*)) is called the information feature vector of hesitant fuzzy element *h*, which is marked as

(6) $$\begin{array}{c} h=\left(s\left(h\right),E\left(h\right)\right), \end{array}$$

where *s*(*h*)=(1/*l*)∑_*j*=1_^*l*^*γ*_*j*_ represents the size of a hesitant fuzzy element and *E*(*h*) is the entropy of hesitant fuzzy element *h*, representing its degree of uncertainty, calculated by Definition 3.1.

The number of elements contained in different hesitant fuzzy elements may be different. In order to facilitate sorting and measurement, it is usually necessary to add elements artificially, which will inevitably destroy the original structure of the data and change the data information. The hesitant fuzzy element is proposed by the information feature vector to solve this kind of problem. The following formulas of the measure and similarity measure of hesitation fuzzy distance based on the feature vector of hesitant fuzzy information are given.

The feature vector of hesitant fuzzy information is to describe the information feature of the hesitant fuzzy element from two different factors, so the dimensions of different components are different; at the same time, there is obviously a correlation between the two components. Therefore, it is not appropriate to choose the traditional distance formula to measure the difference between the two hesitant fuzzy elements. This paper defines a new measure of distance and similarity from the angle of information theory.

**Definition 7 .**

Set *X*={*x*_1_, *x*_2_,…, *x*_*m*_} as a nonempty domain; *A*_*j*_={<*x*_*i*_, *h*_*A*_*j*__(*x*_*i*_) > ∣*x*_*i*_ ∈ *X*}, *j*=1,2, is the two hesitant fuzzy sets defined on *X*, and its information feature vectors are separately *A*_*j*_={(*s*_*A*_*j*__(*x*_*i*_), *E*_*A*_*j*__(*x*_*i*_))∣*x*_*i*_ ∈ *X*}, *j*=1,2. For the convenience of writing, note (*s*_*A*_*j*__(*x*_*i*_), *E*_*A*_*j*__(*x*_*i*_))=(*s*_*ij*_, *E*_*ij*_)=*A*_*j*_(*x*_*i*_), then call

(7) $$\begin{array}{c} d\left(A_{1},A_{2}\right)=R\left(A_{1},A_{2}\right)+R\left(A_{2},A_{1}\right), \end{array}$$

as the distance measure of *A*_1_, *A*_2_. Among which,

(8) $$\begin{array}{c} R\left(A_{1},A_{2}\right)=\frac{1}{m}\overset{m}{\underset{i=1}{\sum }}\left(\underset{\Delta =s,E}{\sum }\left[\Delta_{i1}\cdot \log\frac{\Delta_{i1}}{\Delta_{i2}}+\left(1-\Delta_{i1}\right)\cdot \log\frac{1-\Delta_{i1}}{1-\Delta_{i2}}\right]\right),\\ R\left(A_{2},A_{1}\right)=\frac{1}{m}\overset{m}{\underset{i=1}{\sum }}\left(\underset{\Delta =s,E}{\sum }\left[\Delta_{i2}\cdot \log\frac{\Delta_{i2}}{\Delta_{i1}}+\left(1-\Delta_{i2}\right)\cdot \log\frac{1-\Delta_{i2}}{1-\Delta_{i1}}\right]\right), \end{array}$$

where Δ is the symbolic variable, Δ ∈ {*s*, *E*}.

The distance measure based on the information feature vector is based on the relative entropy idea, and it is easy to verify that it satisfies the following properties.

**Property 2 .**

Set *X*={*x*_1_, *x*_2_,…, *x*_*m*_} as a nonempty domain; *A*_*j*_={<*x*_*i*_, *h*_*A*_*j*__(*x*_*i*_) > ∣*x*_*i*_ ∈ *X*}, *j*=1,2, is the two hesitant fuzzy sets defined on *X*, and its distance measure is *d*(*A*_1_, *A*_2_), then

1. *d*(*A*_1_, *A*_2_) ≥ 0
2. *d*(*A*_1_, *A*_2_)=0, if and only if *A*_1_(*x*_*i*_)=*A*_2_(*x*_*i*_), *i*=1,2,…, *m*
3. *d*(*A*_1_, *A*_2_)=*d*(*A*_2_, *A*_1_)

**Proof**

Make*f*(*t*)=−log *t*; according to*f*^″^(*t*)=(1/*t*^2^) > 0, *f*(*t*) is the concave function in a defined domain, that is, *f*(*λ*_1_*t*_1_+*λ*_2_*t*_2_) ≤ *λ*_1_*f*(*t*_1_)+*λ*_2_*f*(*t*_2_), among which, *λ*_1_, *λ*_2_ ∈ (0,1) and *λ*_1_+*λ*_2_=1. If and only if *t*_1_=*t*_2_, the equal sign is established. Suppose *t*_1_=(Δ_*i*2_/Δ_*i*1_), *t*_2_=(1 − Δ_*i*2_/1 − Δ_*i*1_), *λ*_1_=Δ_*i*1_, *λ*_2_=1 − Δ_*i*1_, bring in *f*(*λ*_1_*t*_1_+*λ*_2_*t*_2_) ≤ *λ*_1_*f*(*t*_1_) +*λ*_2_*f*(*t*_2_) and get

(9) $$\begin{array}{c} 0\leq \Delta_{i1}\cdot \log\frac{\Delta_{i1}}{\Delta_{i2}}+\left(1-\Delta_{i1}\right)\cdot \log\frac{1-\Delta_{i1}}{1-\Delta_{i2}}. \end{array}$$

If and only if when *t*_1_=(Δ_*i*2_/Δ_*i*1_)=(1 − Δ_*i*2_/1 − Δ_*i*1_)=*t*_2_, Δ_*i*1_ · log(Δ_*i*1_/Δ_*i*2_)+(1 − Δ_*i*1_) · log(1 − Δ_*i*1_/1 − Δ_*i*2_)=0; at this moment, Δ_*i*1_=Δ_*i*2_. Then, *R*(*A*_1_, *A*_2_)=(1/*m*)∑_*i*=1_^*m*^(∑_Δ=*s*,*E*_[Δ_*i*1_ · log(Δ_*i*1_/Δ_*i*2_)+(1 − Δ_*i*1_) · log(1 − Δ_*i*1_/1 − Δ_*i*2_)])≥0; if and only if when Δ_*i*1_=Δ_*i*2_(Δ=*s*, *E*, *i*=1,2,…, *m*), *R*(*A*_1_, *A*_2_)=0.

In the same way, we can get *R*(*A*_2_, *A*_1_) ≥ 0; if and only if when Δ_*i*1_=Δ_*i*2_(Δ=*s*, *E*, *i*=1,2,…, *m*), *R*(*A*_2_, *A*_1_)=0. In summary, (1) and (2) can be established.

According to the expression itself, it can be judged that (3) is clearly established.

***Note*.**

Property (2), *A*_1_(*x*_*i*_)=*A*_2_(*x*_*i*_), *i*=1,2,…, *m*, is not equivalent to *A*_1_=*A*_2_; for example, take *A*_1_={<*x*, {0.5}>} and *A*_2_={<*x*, {0,1}>}, it is obvious that *A*_1_ ≠ *A*_2_. The information feature vector is represented as *A*_1_={<*x*, (0.5, 1)>} and *A*_2_={<*x*, {0.5, 1}>}, respectively, according to property (2), and then *d*(*A*_1_, *A*_2_)=0. At this moment, the result is consistent with human intuition because completely ambiguous information and completely contradictory information can convey the same amount of information. This is also the main difference between the distance measure proposed in this paper and other hesitant fuzzy distance measures.

Inspired by TOPSIS, the hesitant fuzzy similarity measure formula based on hesitant fuzzy distance measure is given below.

**Definition 8 .**

Let *X*={*x*_1_, *x*_2_,…, *x*_*m*_} as a given nonempty domain; *j*=1,2 is the two hesitant fuzzy sets defined on *X*, and *j*=1,2 modified to *A*_1_ and *A*_2_.

(10) $$\begin{array}{c} S\left(A_{1},A_{2}\right)=\frac{d\left(A_{1},A_{2}^{c}\right)}{d\left(A_{1},A_{2}\right)+d\left(A_{1},A_{2}^{c}\right)}, \end{array}$$

as the similarity measures of *A*_1_, *A*_2_.

**Property 3 .**

Set *X*={*x*_1_, *x*_2_,…, *x*_*m*_} as a nonempty domain; *A*_*j*_={<*x*_*i*_, *h*_*A*_*j*__(*x*_*i*_) > ∣*x*_*i*_ ∈ *X*}, *j*=1,2, is the two hesitant fuzzy sets defined on *X*, and the similarity measure *A*_1_, *A*_2_ of is *S*(*A*_1_, *A*_2_):

1. 0 ≤ *S*(*A*_1_, *A*_2_) ≤ 1
2. *S*(*A*_1_, *A*_2_)=*S*(*A*_2_, *A*_1_)
3. *S*(*A*_1_, *A*_2_)=0, if and only if when *A*_1_(*x*_*i*_)=*A*_2_^*c*^(*x*_*i*_), *i*=1,2,…, *m*
4. *S*(*A*_1_, *A*_2_)=1, if and only if when *A*_1_(*x*_*i*_)=*A*_2_(*x*_*i*_), *i*=1,2,…, *m*
5. If and only if when *d*(*A*_1_, *A*_2_)=*d*(*A*_1_, *A*_2_^*c*^), *S*(*A*_1_, *A*_2_)=(1/2)

Property 4.2 can be determined by the formula itself. The proof process is omitted.

In practical application, different elements in set *X* have different status and should be given different weights. A similarity measure formula considering weights is given below:

(11) $$\begin{array}{c} S_{w}\left(A_{1},A_{2}\right)=\frac{d_{w}\left(A_{1},A_{2}^{c}\right)}{d_{w}\left(A_{1},A_{2}\right)+d_{w}\left(A_{1},A_{2}^{c}\right)}, \end{array}$$

among which *d*_*w*_(*A*_1_, *A*_2_)=*R*_*w*_(*A*_1_, *A*_2_)+*R*_*w*_(*A*_2_, *A*_1_)=∑_*i*=1_^*m*^(*w*_*i*_∑_Δ=*s*,*E*_[Δ_*i*1_ · log(Δ_*i*1_/Δ_*i*2_)+(1 − Δ_*i*1_) · log((1 − Δ_*i*1_)/(1 − Δ_*i*2_))])+∑_*i*=1_^*m*^(*w*_*i*_∑_Δ=*s*,*E*_[Δ_*i*2_ · log(Δ_*i*2_/Δ_*i*1_)+(1 − Δ_*i*2_) · log((1 − Δ_*i*2_)/(1 − Δ_*i*1_))]). *w*_*i*_ is the weight of element *x*_*i*_(*i*=1,2,…, *m*) and satisfies ∑_*i*=1_^*m*^*w*_*i*_=1, *w*_*i*_ ∈ [0,1]. Obviously, when *w*_*i*_=(1/*m*)(*i*=1,2,…, *m*), *d*_*w*_(*A*_1_, *A*_2_)=*d*(*A*_1_, *A*_2_) and *S*_*w*_(*A*_1_, *A*_2_)=*S*(*A*_1_, *A*_2_).

## 5. Network Clustering Method Based on Hesitant Fuzzy Similarity Measure

The network clustering [33] method is a common method in data clustering analysis, and it is also the best choice to extend the clustering method to the fuzzy environment. The specific process is as follows: the similarity coefficient matrix *P* is constructed by the data similarity measure, and then the cutting level *λ* ∈ [0,1] is selected as *λ*−truncated matrix *P*_*λ*_ of *P*, and replace the principal diagonal element with the scheme symbol. In the lower left of the principal diagonal, the symbol “∗” is used instead of “1” to remove the “0” element. The position of the symbol “∗” is called the node. The so called network is to cross the nodes as the latitude and longitude lines and tie the scheme corresponding to the latitude and longitude lines at the nodes to achieve classification. The main advantage is that the clustering results can be obtained quickly and effectively by using the similarity coefficient matrix directly on the table. The method of clustering analysis in the hesitant fuzzy environment is given below. The calculation process is as follows:

1. Let *A*={*A*_1_, *A*_2_,…, *A*_*m*_} be the set of object to be classified, *F*={*F*_1_, *F*_2_,…, *F*_*n*_} be the decision factor set, and *W*=(*w*_1_, *w*_2_,…,*w*_*n*_)^T^ be the decision factor weight vector. The decision expert group measures the classified objects according to the decision factors and obtains the hesitant fuzzy decision matrix *D*=(*h*_*ij*_)_*m*×*n*_.
2. According to formula (6), the hesitant fuzzy value *h*_*ij*_ is expressed by the information feature vector, and then the decision matrix *D* is transformed into the information feature vector matrix.
3. Calculate the hesitant fuzzy similarity coefficient matrix *P*=(*S*_*w*_(*A*_*i*_, *A*_*k*_))_*m*×*m*_ by using formula (11).
4. Remove elements above the principal diagonal and replace the principal diagonal element with the scheme symbol.
5. Select cutting level *λ* ∈ [0,1] as the *λ*−truncated matrix *P*_*λ*_ of *P*, in the lower left of the principal diagonal, the symbol “∗” is replaced by “1,” and the “0” element is removed. The position of the symbol “∗” is called a node, the node is the latitude and longitude lines, and the node is over the node. The schemes corresponding to the latitude and longitude lines are bundled into one category.

## 6. Illustrative Example

In order to facilitate comparative analysis, this paper uses an example from the literature [32]. Through four factors (price *F*_1_, function *F*_2_, after-sales service *F*_3_, and quality *F*_4_), 7 cell phones *A*_*i*_(*i*=1,2,…, 7) are classified. Assume the factor weight vector is *w*=(0.3, 0.25, 0.2, 0.25)^T^. The decision group gives the evaluation value of mobile phone *A*_*i*_ under the decision factor *F*_*j*_, which is represented by the hesitant fuzzy set *A*_*i*_={*h*_*ij*_ | *j*=1,2,3,4}, (*i*=1,2,…, 7), among which *h*_*ij*_ indicates the degree to which the mobile *A*_*i*_ satisfies the decision factor *F*_*j*_. Then the decision information can be represented by the decision matrix *D*=(*h*_*ij*_)_7×4_ (Table 2). According to the network clustering method, cluster analysis is performed on 7 mobile phones as shown in Table 2.

**Step 1 .**

See Table 2.

**Step 2 .**

According to the formula (6), the hesitation fuzzy value in the hesitating fuzzy decision matrix is transformed into the information feature vector matrix (Table 3).

For example, the data (0.5, 0.68) in the first column of the first row in Table 3 are the information feature vector corresponding to the data {0.4, 0.6} in the first column of the first row in Table 2. They are calculated according to formula (6), where 0.5=(1/2)(0.4+0.6), 0.68={(1/2) · [(1 − 2  · |0.4 − 0.5|)+(1 − 2 · |0.6 − 0.5|)]}^2^+(|0.4 − 0.6|)^2^.

**Step 3 .**

Calculate hesitant fuzzy similarity coefficient matrix by using formula (4):

(12) $$\begin{array}{c} P=\left[\begin{array}{ccccccc} 1 & 0.6183 & 0.6168 & 0.4298 & 0.5121 & 0.4077 & 0.3696\\ 0.6183 & 1 & 0.9292 & 0.5020 & 0.3064 & 0.4434 & 0.1985\\ 0.6168 & 0.9292 & 1 & 0.4927 & 0.2484 & 0.4549 & 0.0793\\ 0.4298 & 0.5020 & 0.4927 & 1 & 0.4934 & 0.5653 & 0.5139\\ 0.5121 & 0.3064 & 0.2484 & 0.4934 & 1 & 0.5169 & 0.7172\\ 0.4077 & 0.4434 & 0.4549 & 0.5653 & 0.5169 & 1 & 0.5401\\ 0.3696 & 0.1985 & 0.0793 & 0.5139 & 0.7172 & 0.5401 & 1 \end{array}\right]. \end{array}$$

The first row and second column data 0.6183 are the similarity measurement between the date in the first row and the date in the second row in Table 3 and is calculated according to formula (11):

(13) $$\begin{array}{c} d_{w}\left(A_{1},A_{2}\right)=0.3\cdot \left[0.5\cdot \log\frac{0.5}{0.33}+\left(1-0.5\right)\cdot \log\frac{1-0.5}{1-0.33}+0.68\cdot \log\frac{0.68}{0.48}+\left(1-0.68\right)\cdot \log\frac{1-0.68}{1-0.48}+0.33\cdot \log\frac{0.33}{0.5}+\left(1-0.33\right)\cdot \log\frac{1-0.33}{1-0.5}+0.48\cdot \log\frac{0.48}{0.68}+\left(1-0.48\right)\cdot \log\frac{1-0.48}{1-0.68}\right]\\ +0.25\cdot \left[0.47\cdot \log\frac{0.47}{0.3}+\left(1-0.47\right)\cdot \log\frac{1-0.47}{1-0.3}+0.68\cdot \log\frac{0.68}{0.4}+\left(1-0.68\right)\cdot \log\frac{1-0.68}{1-0.4}+0.3\cdot \log\frac{0.3}{0.47}+\left(1-0.3\right)\cdot \log\frac{1-0.3}{1-0.47}+0.68\cdot \log\frac{0.68}{0.4}+\left(1-0.68\right)\cdot \log\frac{1-0.68}{1-0.4}\right]\\ +0.2\cdot \left[0.25\cdot \log\frac{0.25}{0.37}+\left(1-0.25\right)\cdot \log\frac{1-0.25}{1-0.37}+0.26\cdot \log\frac{0.26}{0.57}+\left(1-0.26\right)\cdot \log\frac{1-0.26}{1-0.57}+0.37\cdot \log\frac{0.37}{0.25}+\left(1-0.37\right)\cdot \log\frac{1-0.37}{1-0.25}+0.57\cdot \log\frac{0.57}{0.26}+\left(1-0.57\right)\cdot \log\frac{1-0.57}{1-0.26}\right]\\ +0.25\cdot \left[0.4\cdot \log\frac{0.4}{0.35}+\left(1-0.4\right)\cdot \log\frac{1-0.4}{1-0.35}+0.64\cdot \log\frac{0.64}{0.5}+\left(1-0.64\right)\cdot \log\frac{1-0.64}{1-0.5}+0.35\cdot \log\frac{0.35}{0.4}+\left(1-0.35\right)\cdot \log\frac{1-0.35}{1-0.4}+0.5\cdot \log\frac{0.5}{0.64}+\left(1-0.5\right)\cdot \log\frac{1-0.5}{1-0.64}\right]\\ =0.3169,\\ d_{w}\left(A_{1},A_{2}^{c}\right)=0.3\cdot \left[0.5\cdot \log\frac{0.5}{0.67}+\left(1-0.5\right)\cdot \log\frac{1-0.5}{1-0.67}+0.68\cdot \log\frac{0.68}{0.48}+\left(1-0.68\right)\cdot \log\frac{1-0.68}{1-0.48}+0.67\cdot \log\frac{0.67}{0.5}+\left(1-0.67\right)\cdot \log\frac{1-0.67}{1-0.5}+0.48\cdot \log\frac{0.48}{0.68}+\left(1-0.48\right)\cdot \log\frac{1-0.48}{1-0.68}\right]\\ +0.25\cdot \left[0.47\cdot \log\frac{0.47}{0.7}+\left(1-0.47\right)\cdot \log\frac{1-0.47}{1-0.7}+0.68\cdot \log\frac{0.68}{0.4}+\left(1-0.68\right)\cdot \log\frac{1-0.68}{1-0.4}+0.7\cdot \log\frac{0.7}{0.47}+\left(1-0.7\right)\cdot \log\frac{1-0.7}{1-0.47}+0.68\cdot \log\frac{0.68}{0.4}+\left(1-0.68\right)\cdot \log\frac{1-0.68}{1-0.4}\right]\\ +0.2\cdot \left[0.25\cdot \log\frac{0.25}{0.63}+\left(1-0.25\right)\cdot \log\frac{1-0.25}{1-0.63}+0.26\cdot \log\frac{0.26}{0.57}+\left(1-0.26\right)\cdot \log\frac{1-0.26}{1-0.57}+0.63\cdot \log\frac{0.63}{0.25}+\left(1-0.63\right)\cdot \log\frac{1-0.63}{1-0.25}+0.57\cdot \log\frac{0.57}{0.26}+\left(1-0.57\right)\cdot \log\frac{1-0.57}{1-0.26}\right]\\ +0.25\cdot \left[0.4\cdot \log\frac{0.4}{0.65}+\left(1-0.4\right)\cdot \log\frac{1-0.4}{1-0.65}+0.64\cdot \log\frac{0.64}{0.5}+\left(1-0.64\right)\cdot \log\frac{1-0.64}{1-0.5}+0.65\cdot \log\frac{0.65}{0.4}+\left(1-0.65\right)\cdot \log\frac{1-0.65}{1-0.4}+0.5\cdot \log\frac{0.5}{0.64}+\left(1-0.5\right)\cdot \log\frac{1-0.5}{1-0.64}\right]\\ =0.5134. \end{array}$$

**Step 4 .**

Remove elements above the principal diagonal, and replace the principal diagonal element with the scheme symbol, that is:

(14) $$\begin{array}{c} P=\left[\begin{array}{ccccccc} A_{1} & & & & & & \\ 0.6183 & A_{2} & & & & & \\ 0.6168 & 0.9292 & A_{3} & & & & \\ 0.4298 & 0.5020 & 0.4927 & A_{4} & & & \\ 0.5121 & 0.3064 & 0.2484 & 0.4934 & A_{5} & & \\ 0.4077 & 0.4434 & 0.4549 & 0.5653 & 0.5169 & A_{6} & \\ 0.3696 & 0.1985 & 0.0793 & 0.5139 & 0.7172 & 0.5401 & A_{7} \end{array}\right]. \end{array}$$

**Step 5 .**

Select the cutting level *λ* ∈ [0,1] as the*λ*−truncation matrix *P*_*λ*_ of *P* and then classify through the network:

1. When 0.9292 < *λ* ≤ 1, they are divided into 7 categories: {*A*_1_}, {*A*_2_}, {*A*_3_}, {*A*_4_}, {*A*_5_}, {*A*_6_}, {*A*_7_}
2. When 0.7172 < *λ* ≤ 0.9291, they are divided into 6 categories: {*A*_1_}, {*A*_2_, *A*_3_}, {*A*_4_}, {*A*_5_}, {*A*_6_}, {*A*_7_}
3. When 0.6183 < *λ* ≤ 0.7172, they are divided into 5 categories: {*A*_1_}, {*A*_2_, *A*_3_}, {*A*_4_}, {*A*_5_, *A*_7_}, {*A*_6_}
4. When 0.5653 < *λ* ≤ 0.6183, they are divided into 4 categories: {*A*_1_, *A*_2_, *A*_3_}, {*A*_4_}, {*A*_5_, *A*_7_}, {*A*_6_}
5. When 0.5401 < *λ* ≤ 0.5653, they are divided into 3 categories: {*A*_1_, *A*_2_, *A*_3_}, {*A*_4_, *A*_6_}, {*A*_5_, *A*_7_}
6. When 0.4927 < *λ* ≤ 0.5401, they are divided into 2 categories: {*A*_1_, *A*_2_, *A*_3_}, {*A*_4_, *A*_5_, *A*_6_, *A*_7_}
7. When 0 ≤ *λ* ≤ 0.4927, they are divided into 1 category: {*A*_1_, *A*_2_, *A*_3_, *A*_4_, *A*_5_, *A*_6_, *A*_7_}

Next, the clustering results of this paper are compared with those of the literature [28], literature [29], literature [31], and literature [32], and the results are analyzed. The clustering results obtained by other methods are shown in Table 4.

Different literatures choose different measures to measure the degree of closeness between samples; among them, the literature [28] is based on the correlation coefficient; the literature [29] and literature [31] are based on the distance measure; and the literature [32] and this paper are based on the similarity measure. In order to compare the sensitivity of various measures, it is necessary to analyze the variance of the measurement data. The larger the variance, the higher the sensitivity of the corresponding measure. The results are shown in Table 5. Furthermore, in order to compare the effectiveness of various methods, the D-B index [34] of the clustering results is calculated separately. The distance measure selected for calculating the D-B index of the literature [28] is *d*(*A*, *B*)=1 − *ρ*(*A*, *B*). The results are shown in Table 5.

It can be found from Table 5 that (1) the similarity measure proposed in this paper has higher sensitivity than other measures and the clustering result has better robustness and (2) tThe D-B index of this paper is smaller, indicating that the clustering results are better.

Through comparison, it can be found that (1) the results obtained by using the method proposed in this paper are consistent with those obtained in the literature [32], which to some extent reflects the effectiveness of the method proposed in this paper; (2) the results of the classification in the literature [28] are not precise and accurate, as it is intuitively possible to judge from the data in Table 2*S*(*A*_2_, *A*_3_) > *S*(*A*_2_, *A*_4_), so it is more appropriate to classify them *A*_2_, *A*_3_ as a group; (3) the methods of the literature [29] and literature [31] are put forward based on the distance formula in the literature [4], but the resolution of the distance formula is not high, and the results are sometimes inconsistent with the facts [32], which will inevitably affect the classification results; (4) the data must be processed by equal length in the literature [28], literature [29], literature [31], and literature [32], which will inevitably affect the clustering results. The reason why the results in this paper are consistent with the literature [32] is that the difference in the number of hesitant fuzzy numbers in Table 2 is small, and the size of each element in the same hesitant fuzzy number is not much different, and if not so, the results must be different from those obtained by the present method.

## 7. Conclusion

In this paper, the hesitant fuzzy information feature vector is used as the entry point, which provides a new idea for solving various hesitant fuzzy measures. Then the hesitant fuzzy uncertainty measure, distance measure, and similarity measure are studied. Finally, a clustering method for fuzzy information is proposed. Through analyzing the results of the example, it has been proved that this method is faster and more effective in practical applications. The main contributions of this paper are (1) it effectively avoids the problem of processing data with equal length in the research of the measure of hesitant fuzzy set and (2) combined with the similarity measure proposed by TOPSIS idea, the resolution between schemes can be improved. Subsequent research on hesitant fuzzy set theory and application based on hesitant fuzzy information feature vectors will be a meaningful topic.

## Figures and Tables

**Table 1:** Comparison table of hesitant fuzzy entropy values.

*E*(*h*)	*h*_1_	*h*_2_	*h*_3_	*h*_4_	*h*_5_	*h*_6_	*h*_7_	*h*_8_
*E*_Xu_(*h*)	0.881	0.971	0.640	1	0.993	0.993	0.995	0.995
*E*_Pa_(*h*)	0.6	0.8	0.4	0.867	0.7	0.9	0.8	0.6
*E*_Lv_(*h*)	0.4	0.68	0.177	0.774	0.58	0.82	0.68	0.556

**Table 2:** Hesitation fuzzy decision matrix.

	*F*_1_	*F*_2_	*F*_3_	*F*_4_
*A*_1_	{0.4, 0.6}	{0.3, 0.5, 0.6}	{0.2, 0.3}	{0.4}
*A*_2_	{0.2, 0.3, 0.5}	{0.2, 0.4}	{0.2, 0.4, 0.5}	{0.3, 0.4}
*A*_3_	{0.2, 0.3, 0.4}	{0.2, 0.4}	{0.2, 0.3, 0.5}	{0.3}
*A*_4_	{0.3, 0.45, 0.75}	{0.3, 0.6}	{0.3, 0.6, 0.75}	{0.45, 0.6}
*A*_5_	{0.8}	{0.4, 0.6, 0.7}	{0.3, 0.4}	{0.6, 0.8}
*A*_6_	{0.4, 0.5, 0.7}	{0.4, 0.6}	{0.4, 0.6, 0.7}	{0.5, 0.6}
*A*_7_	{0.6, 0.7, 0.9}	{0.6, 0.8}	{0.6, 0.8, 0.9}	{0.7, 0.8}

**Table 3:** Information feature vector matrix.

	*F*_1_	*F*_2_	*F*_3_	*F*_4_
*A*_1_	(0.5, 0.68)	(0.47, 0.68)	(0.25, 0.26)	(0.4, 0.64)
*A*_2_	(0.33, 0.48)	(0.3, 0.4)	(0.37, 0.57)	(0.35, 0.5)
*A*_3_	(0.3, 0.38)	(0.3, 0.4)	(0.33, 0.48)	(0.3, 0.36)
*A*_4_	(0.5, 0.53)	(0.45, 0.58)	(0.55, 0.49)	(0.53, 0.75)
*A*_5_	(0.8, 0.16)	(0.57, 0.57)	(0.35, 0.5)	(0.7, 0.4)
*A*_6_	(0.53, 0.68)	(0.5, 0.68)	(0.57, 0.58)	(0.55, 0.82)
*A*_7_	(0.73, 0.32)	(0.7, 0.4)	(0.77, 0.26)	(0.75, 0.26)

**Table 4:** Clustering result comparison table.

Category	Literature [28] method	Literature [29] method	Literature [31] method	Literature [32] method
1	{*A*_1_, *A*_2_, *A*_3_, *A*_4_, *A*_5_, *A*_6_, *A*_7_}	{*A*_1_, *A*_2_, *A*_3_, *A*_4_, *A*_5_, *A*_6_, *A*_7_}	{*A*_1_, *A*_2_, *A*_3_, *A*_4_, *A*_5_, *A*_6_, *A*_7_}	{*A*_1_, *A*_2_, *A*_3_, *A*_4_, *A*_5_, *A*_6_, *A*_7_}
2		{*A*_1_, *A*_2_, *A*_3_, *A*_4_, *A*_5_, *A*_6_}, {*A*_7_}	{*A*_1_, *A*_2_, *A*_3_, *A*_4_, *A*_6_, *A*_7_}, {*A*_5_}	{*A*_1_, *A*_2_, *A*_3_}, {*A*_4_, *A*_5_, *A*_6_, *A*_7_}
3	{*A*_1_, *A*_5_}, {*A*_2_, *A*_3_, *A*_4_}, {*A*_6_, *A*_7_}	{*A*_1_, *A*_2_, *A*_3_, *A*_4_, *A*_6_}, {*A*_5_}, {*A*_7_}	{*A*_1_, *A*_2_, *A*_3_, *A*_4_, *A*_6_}, {*A*_5_}, {*A*_7_}	{*A*_1_, *A*_2_, *A*_3_}, {*A*_4_, *A*_6_}, {*A*_5_, *A*_7_}
4	{*A*_1_}, {*A*_2_, *A*_3_, *A*_4_}, {*A*_5_}, {*A*_6_, *A*_7_}	{*A*_1_, *A*_4_, *A*_6_}, {*A*_2_, *A*_3_}, {*A*_5_}, {*A*_7_}	{*A*_1_, *A*_2_, *A*_3_}, {*A*_4_, *A*_6_}, {*A*_5_}, {*A*_7_}	{*A*_1_, *A*_2_, *A*_3_}, {*A*_4_}{*A*_5_, *A*_7_}, {*A*_6_}
5	{*A*_1_}, {*A*_2_, *A*_4_}, {*A*_3_}, {*A*_5_}, {*A*_6_, *A*_7_}	{*A*_1_}, {*A*_2_, *A*_3_}, {*A*_4_, *A*_6_}, {*A*_5_}, {*A*_7_}	{*A*_1_}, {*A*_2_, *A*_3_}, {*A*_4_, *A*_6_}, {*A*_5_}, {*A*_7_}	{*A*_1_}, {*A*_2_, *A*_3_}, {*A*_4_}, {*A*_5_, *A*_7_}, {*A*_6_}
6	{*A*_1_}, {*A*_2_, *A*_4_}, {*A*_3_}, {*A*_5_}, {*A*_6_}, {*A*_7_}	{*A*_1_}, {*A*_2_, *A*_3_}, {*A*_4_}, {*A*_5_}, {*A*_6_, *A*_7_}	{*A*_1_}, {*A*_2_, *A*_3_}, {*A*_4_}, {*A*_5_}, {*A*_6_, *A*_7_}	{*A*_1_}, {*A*_2_, *A*_3_}, {*A*_4_}, {*A*_5_}, {*A*_6_}, {*A*_7_}
7		{*A*_1_}, {*A*_2_}, {*A*_3_}, {*A*_4_}, {*A*_5_}, {*A*_6_}, {*A*_7_}	{*A*_1_}, {*A*_2_}, {*A*_3_}, {*A*_4_}, {*A*_5_}, {*A*_6_}, {*A*_7_}	{*A*_1_}, {*A*_2_}, {*A*_3_}, {*A*_4_}, {*A*_5_}, {*A*_6_}, {*A*_7_}

**Table 5:** Variance comparison table.

	Literature [28]	Literature [29]	Literature [31]	Literature [32]	This paper
Variance	0.0038	0.0152	0.0152	0.0319	0.0328
D-B index	1.7652	0.9236	0.9983	0.7326	0.6537

## Data Availability

Previously reported data were used to support this study and are available at DOI: 10.1080/00207721.2013.797073. These prior studies (and datasets) are cited at relevant places within the text as references [29].
